# Performance of the Emotiv Epoc headset for P300-based applications

**DOI:** 10.1186/1475-925X-12-56

**Published:** 2013-06-25

**Authors:** Matthieu Duvinage, Thierry Castermans, Mathieu Petieau, Thomas Hoellinger, Guy Cheron, Thierry Dutoit

**Affiliations:** 1 TCTS Lab, University of Mons, 20, Place du Parc, Mons 7000, Belgium; 2 LNMB Lab, Université Libre de Bruxelles, 50 Av F Roosevelt CP 168, Brussels, Belgium

**Keywords:** Brain-computer interfaces, Emotiv Epoc headset, Gait, Hardware comparative study, Low-cost, P300

## Abstract

**Background:**

For two decades, EEG-based Brain-Computer Interface (BCI) systems have been widely studied in research labs. Now, researchers want to consider out-of-the-lab applications and make this technology available to everybody. However, medical-grade EEG recording devices are still much too expensive for end-users, especially disabled people. Therefore, several low-cost alternatives have appeared on the market. The Emotiv Epoc headset is one of them. Although some previous work showed this device could suit the customer’s needs in terms of performance, no quantitative classification-based assessments compared to a medical system are available.

**Methods:**

This paper aims at statistically comparing a medical-grade system, the ANT device, and the Emotiv Epoc headset by determining their respective performances in a P300 BCI using the same electrodes. On top of that, a review of previous Emotiv studies and a discussion on practical considerations regarding both systems are proposed. Nine healthy subjects participated in this experiment during which the ANT and the Emotiv systems are used in two different conditions: sitting on a chair and walking on a treadmill at constant speed.

**Results:**

The Emotiv headset performs significantly worse than the medical device; observed effect sizes vary from medium to large. The Emotiv headset has higher relative operational and maintenance costs than its medical-grade competitor.

**Conclusions:**

Although this low-cost headset is able to record EEG data in a satisfying manner, it should only be chosen for non critical applications such as games, communication systems, etc. For rehabilitation or prosthesis control, this lack of reliability may lead to serious consequences. For research purposes, the medical system should be chosen except if a lot of trials are available or when the Signal-to-Noise Ratio is high. This also suggests that the design of a specific low-cost EEG recording system for critical applications and research is still required.

## Background

Since its beginning, EEG-based non-invasive Brain-Computer Interfaces (BCI) have mainly targeted disabled people thanks to plenty of different applications [1]. Communication and control are some of them, notably by allowing to control a mouse, to use a web browser or to spell words just by thought. Other main research areas are the study of motor substitution or motor rehabilitation whose main applications are hand grasping [2] and wheelchair control [3]. Finally, for healthy end-users, BCIs have also been used to augment interactivity in games by using multimodality from the Electroencephalography (EEG) signals and the standard control [4,5].

Although EEG recording devices are much less expensive and portable than other brain activity recording techniques such as Magnetoencephalography (MEG), Functional Magnetic Resonance Imaging (fMRI), Functional Near Infrared Spectroscopy (fNIRS), they are still much too expensive for a daily use according to a customer point of view, especially for disabled people. This is why plenty of commercial EEG devices are now available such as Neurosky, Mindflex, Emotiv Epoc, etc [6]. According to [6], the best low-cost EEG device in terms of usability is the Emotiv Epoc headset.

However, to our knowledge, no deep scientific quantitative classification-based comparison of this headset with a medical system has been done. Based on a P300/*odd-ball* BCI, the NeuroPhone smartphone application [7] processes EEG signals collected in realtime by the Emotiv Epoc headset. In their paper, the authors present strongly above chance results showing that this device is a good low-cost alternative. However, a comparison of the performance obtained with another EEG acquisition system is not available. Under indoor and outdoor ambulatory conditions, the same conclusion arises for a recent study combining the Emotiv electrodes and an EEG cap in order to get a precise positioning [8]. They did not give the hardware comparative results and the difference with sitting conditions. Furthermore, this way of proceeding could be criticized due to the potential damage to the hardware device. In [9], the authors show that P300 response averaged over around 600 stimuli is similar between the Emotiv system and the Neuroscan system with an inter-class correlation in the [0.7–0.8] range. The mismatch negativity potentials they obtain also show a strong similarity except for noisy potentials (half of the subjects). Basically, it confirms a previous study [10]: data provided by both systems are alike in general, but the signal has a better Signal-to-Noise Ratio (SNR) in the medical system (g.tec device).

Based on a SSVEP BCI in [11], the researchers showed that the Emotiv Epoc headset provides decreased performance compared to an ActiCap system with 8 channels located over the occipital area under sitting conditions. Given the advantage of placing all the electrodes around the region of interest for the SSVEP paradigm, the conclusion of underperformance is misleading as far as this experiment can not show the true potential of the headset. Indeed, the previous experiment mostly compares the ability of the devices to record EEG at the right place for a given BCI paradigm instead of their intrinsic performance. One should keep in mind that electrodes could be reorganized according to the final goal as performed in [8]. However, it gives an indication that the Emotiv Epoc headset is not dedicated to all the available BCI paradigms. In [12], a comparative study with a g.tec device was initiated also showing worse results for the Emotiv Epoc headset. On four sitting subjects, they obtained an underperformance of around 10%, which is coherent with [13]. However, only four common electrodes were used and four subjects were evaluated without considering motion and real life applications.

Finally, in [14], based on mental tasks (relaxation and imaging of two types of pictures), it was reported that an ActiCap medical system was much better than the Emotiv Epoc. However, the authors did not compare the performance of both systems in the same experimental conditions. For instance, the electrode number and their location were significantly different. Consequently, the conclusions of this study are possibly misleading when assessing the actual Emotiv headset performance.

In order to properly study the Emotiv Epoc headset performance compared to a medical device and assess the relevancy of this low-cost headset [15], this paper investigates the P300 paradigm in terms of recognition performance under walking and sitting conditions to detect whether conclusions remain the same or not in such different situations. It also provides a statistical comparative performance under walking and sitting conditions to detect whether those recording devices are resistant to movement artefacts. This paper is an extension of a previous study [13]. The Background section summarizes the main results of the previous Emotiv Epoc headset studies. The Materials and method section details both recording systems, describes the P300 paradigm, the interface and the pipeline and gives the performance measures as well as the statistical approach. In the Results and discussion section, the P300 results are presented and some other practical considerations are given. Finally, the Conclusions section summarizes the paper and provides some future works.

## Materials and method

### Acquisition systems

#### ANT

**Figure 1:**
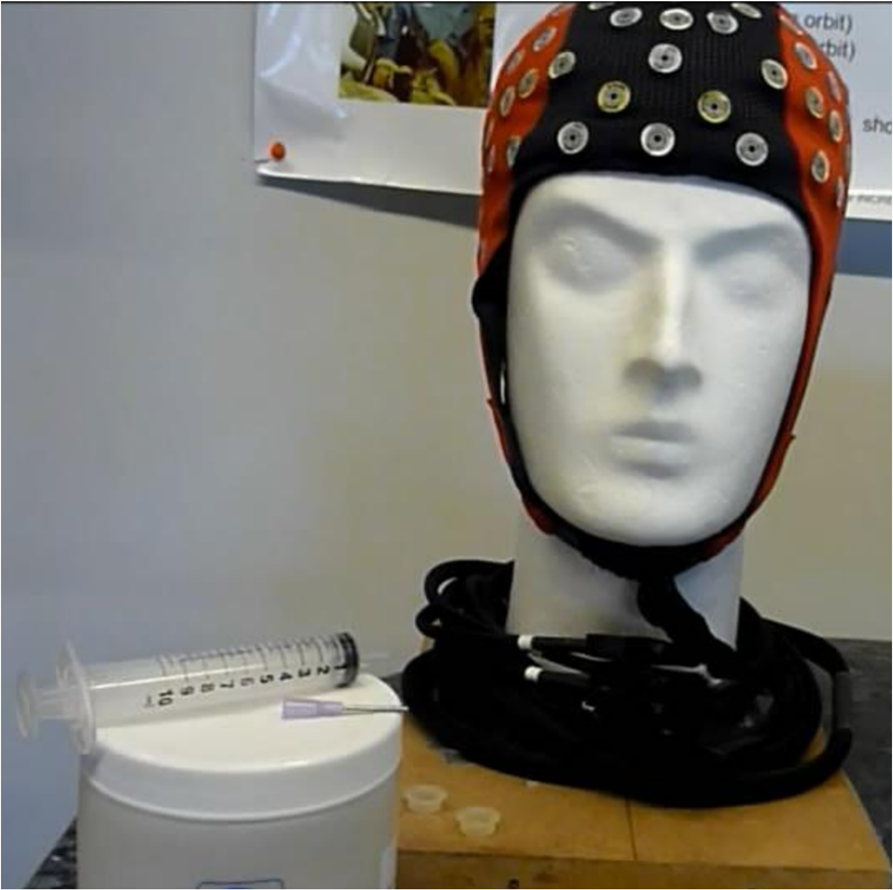
**ANT device.** The cables and the electrodes are well fixed in order to decrease motion artefacts. The cables are shielded to avoid interference.

The ANT acquisition system (Advanced Neuro Technology, ANT, Enschede, The Netherlands) is composed of a high-density WaveGuard cap system and the corresponding full-band DC amplifier. As shown in Figure 1, the WaveGuard cap has 128 Ag/AgCl electrodes covering all the major brain cortical areas (based on the International 10–20 system) with shielded wires in order to be less influenced by exterior noise. Moreover, three different cap sizes are available to adapt as well as possible to the subject’s head specificities. Regarding the full-band DC amplifier, it can reach a sampling rate of 2048 Hz.

In our experiment, electrode impedance was measured and maintained under 20 k *Ω* for each channel using electrode gel and signals were visually checked before recording. The studied electrode positions are the same as provided by the Emotiv Epoc headset. The hardware and software overall cost is around 30,000–50,000<DOLLAR/>.

The ANT device is provided with the ASA software. It is composed of several main tools: pre-processing, Event-Related Potential analysis (ERP), source reconstruction using inverse models and time-frequency analysis. All these aspects allow more advanced users, typically researchers/physicians to deeply study the brain signals.

#### Emotiv EPOC

**Figure 2:**
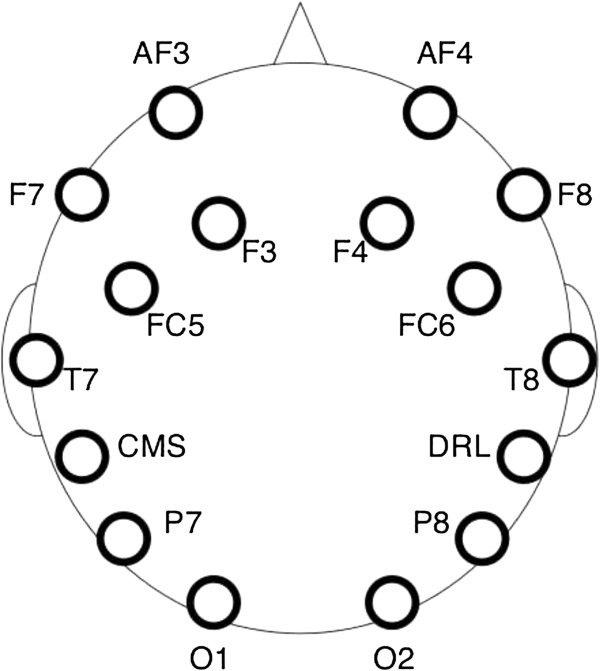
**Emotiv electrodes.** The Emotiv Epoc headset is composed of 14 different electrodes in addition to two references (picture attributed to Emotiv and Emotiv EPOC neuroheadset).

**Figure 3:**
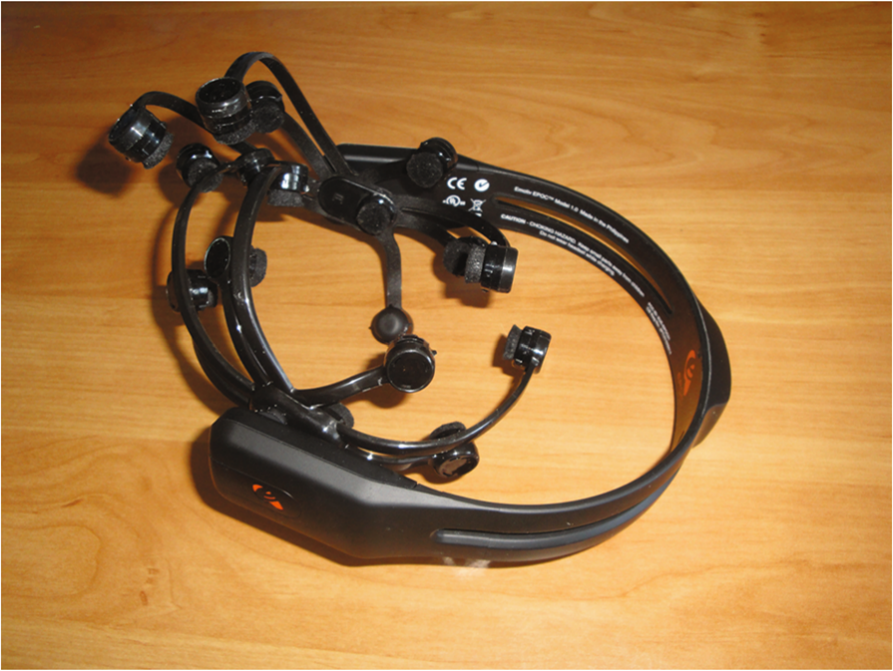
**Emotiv picture.** The Emotiv Epoc headset is handy to place and wireless.

As announced on the Emotiv website, the Emotiv Epoc headset and its Software Development Kit for research mainly includes 14 channels (plus CMS/DRL references, P3/P4 locations) each based on saline sensors. Available channels (also based on the International 10–20 locations) are depicted in Figure 2. This headset has not the ability to cope with all the BCI paradigms with the same success without modifying the hardware. For instance, the motor imagery paradigm, which requires central electrodes, should provide bad performance. As shown in Figure 3, the headset is completely wireless and has a large Lithium-based battery autonomy of 12 hours. The sampling rate can reach 128 Hz. Additionally, gyroscope generates optimal positional information for cursor and camera controls.

In our experiment, all the standard available electrodes of the Emotiv Epoc headset were used. Electrode impedance was decreased by using saline liquid until the level required by the software was reached (in the 10–20 *kΩ* range) and was checked along the experiment. For the research edition, the total cost is 750<DOLLAR/>.

The Emotiv headset is provided with three different detection software: Expressiv, Affectiv and Cognitiv suites. The Expressiv suite aims at interpreting the user’s facial expressions in real-time. The Affectiv suite aims at monitoring the user’s emotional states in real-time. The Cognitiv suite aims at performing standard BCI-like control.

### BCI system

#### P300-based approach

**Figure 4:**
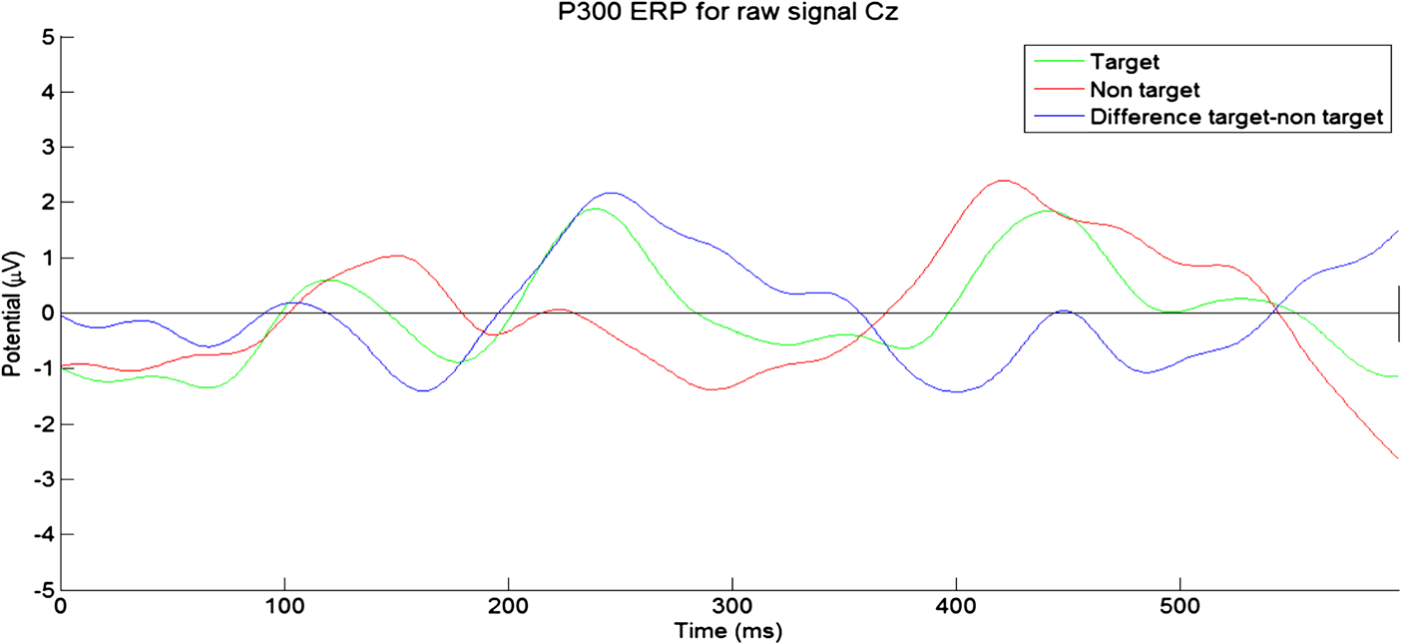
**P300 picture.** When a rare and relevant stimulus appears, a P300 potential is elicited around 250 ms after the stimulus (Target). Otherwise, no particular brain response is observed (Non target). Here, due to our pipeline, another P300 response is elicited. Because the flashes are not strictly rare, the magnitude of the P300 is quite low. Finally, the difference between both conditions is depicted for information. The relatively small magnitude of the P300 peak is likely due to the non-rare occurrence of the target.

As illustrated in Figure 4, the P300 evoked potential is an involuntary positive potential that arises around 300 ms after the user has perceived a relevant and rare stimulus [16]. This is commonly used in an odd-ball paradigm, in which the user is requested to attend to a random sequence composed of two kind of stimuli with one stimulus much less frequent than the other one. If the infrequent stimulus is relevant to the user who is putting his attention on it (e.g. silently counting it), its actual appearance activates a P300 waveform in the user’s EEG, which is mainly located in the parietal areas [17].

**Figure 5:**
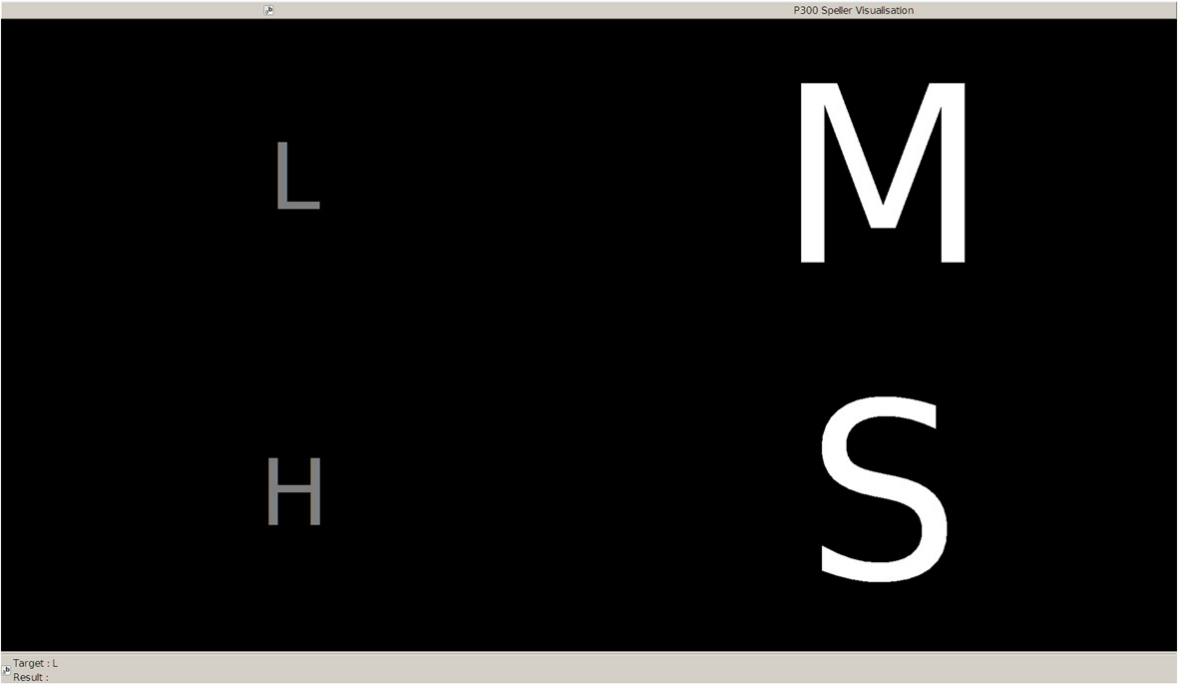
**P300 interface.** P300 visualization is divided into four states. After the target the subject has to look at is highlighted in green, the rows/columns are randomly flashed. By detecting the P300 responses, the system is able to recognize the looked target.

Following previous work [13,18] and inspired from the 6×6 matrix P300-speller text editor [19], we are interested in a four-state BCI as depicted in Figure 5. Indeed, if mobile applications have to be considered (e.g. control of a prosthesis or augmented interaction in daily life), one can not afford using a lot of states.

The recognition of a given state is quite simple. At the beginning of one trial, one of the four letters is highlighted in green. Then, the subject has to look at this letter when each row/column is flashed 12 times to increase the low SNR due to disturbances of other brain, muscular and ocular activities. At the intersection of the detected P300 responses, the computer is able to determine which letter/symbol the subject was looking at.

Obviously, in this approach, the interface is not strictly an odd-ball paradigm. Actually, each letter is flashed 50% of the time, which is not really a rare event. However, previous work showed that this approach provides good results and was thus used in this paper [18].

**Figure 6:**
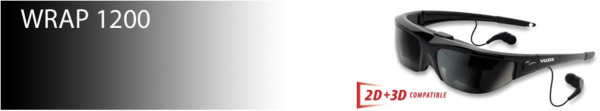
**Vuzix illustration.** The Vuzix eyewear should allow ambulatory P300-based BCI.

For out-of-the-lab applications, the requirement of an external screen to activate stimuli used in this experiment could be problematic. However, thanks to specific emerging and well-designed VUZIX augmented reality eyewears (Vuzix, Rochester, NY, USA), this problem could be circumvented. As shown in Figure 6, by displaying stimuli on a semi-transparent module containing all the key hardware elements, the device should allow ambulatory P300 applications. Again, the tradeoff of four different states represented by a letter at the four corners of the semi-transparent glasses appears to be a more realistic solution.

#### P300 pipeline

**Figure 7:**
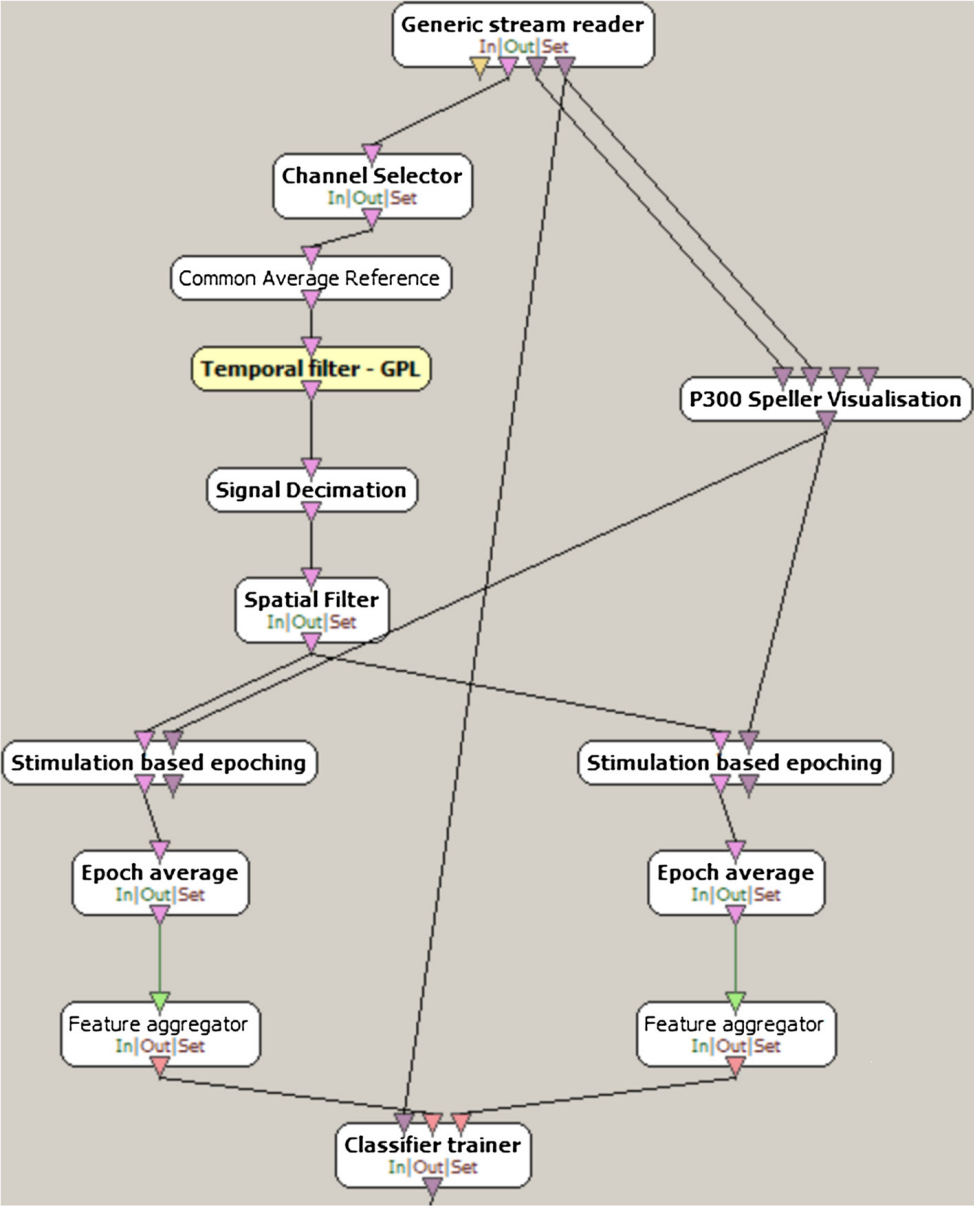
**Pipeline.** The pipeline includes a channel selection, a common average referencing, a high pass filtering, a downsampling, an xDawn spatial filtering, a two-epoch averaging and a LDA classifier (pink flows). To elicit the P300 potential, a rigorous stimulation management (purple flows) has to be done for synchronizing the P300 averaging.

Considering the Emotiv electrodes on the ANT device (using a common average reference for both devices), the pipeline of the approach (shown in Figure 7) includes different main parts: a temporal high-pass filter, an xDAWN-based spatial filter, an epoch averaging and a Linear Discriminant Analysis (LDA) classifier using a voting rule for the final decision. In order to provide more precise coherent comparison results, this is the same pipeline as developed in our conference paper initiating the comparison of both systems [13]. As discussed in [20], gait-related artefact removal techniques do not bring significant better performance and are thus not used in this pipeline. Ocular and muscular artefacts are basically not linked to the P300 task (except the first gaze to the letter) leading to a strongly mitigated effect by the averaging. This procedure was implemented in the open source OpenVibe software [21].

In order to obtain a better performance, the EEG signal is high-pass filtered at 1 Hz using a 4th order Butterworth filter. By trial and error, we observed that downsampling (an anti-aliasing filter to avoid adding noise was used in the OpenVibe downsampling box) the data at 32 Hz allows a better behaviour of the LDA classifier while decreasing observed noise. Indeed, the P300 potential is mainly located below 16 Hz whereas an undesired slow biological drift can interfere with the pipeline [22]. This also removes high-frequency noise such as power line interference.

Afterwards, a spatial filter is designed thanks to the xDAWN algorithm [23]. This algorithm aims at magnifying the P300 response by considering both signal and noise contrary to a common principal component analysis. Target/non-target epochs have to be separately fed in the algorithm. By linearly combining EEG channels, this algorithm defines P300 and noise subspaces. When projecting EEG signals into these subspaces, P300 detection is enhanced. In this paper, three projection components were retained as the authors basically advise to divide the number of channels by four.

Then, as proposed in the Openvibe software, we use a 600 ms time window epoch. The beginning of the epoch is synchronized with the flashed target. In order to obtain a better SNR, groups of two epochs corresponding to a specific row/column are averaged. The flash, no flash and inter-repetition duration are respectively 0.2 s, 0.1 s and 1 s.

Finally, a 12-fold Linear Discriminant Analysis classifier (LDA) is used to detect whether a P300 was elicited in the brain. In the *k*-fold approach, the training set is splitted into *k* uniform groups. Then, *k*−1 groups are used for training the LDA classifier and the test is performed on the remaining group. After performing this *k* times, the classifier obtaining the best results is chosen. The reported *k*-fold value is the average of the *k* training performance. For each two-grouped time window, the output value of the classifier represents the distance to a hyperplane separating at best the target/non-target P300 classes. This value could also be considered as a confident measure. For a given trial, in a voting classifier, the row/column which has been activated is determined by summing six consecutive LDA outputs (12 repetitions) and by choosing the most probable target.

### Performance evaluation

#### Performance measures

In this paper, two performance measures are assessed: *k*-fold classification and test set classification rates. The former measure is the single two-grouped epoch classification accuracy, i.e. without any *temporal* averaging. This measure is obtained only on the training set. This helps to assess the difficulties to learn data due to a different hardware device and could be interpreted as an indirect measure of the SNR by their intrinsic correlation. Indeed, if the SNR is increased, the classification task is made easier leading to enhanced performance. Because a specific care for the Emotiv Epoc electrode positioning was performed, the effect of misalignment should be highly mitigated. Furthermore, the P300 response has an inter-subject distribution variability and thus, the effect should be averaged across subjects.

The test set classification rate introduces an averaging in the decision process. In the P300 pipeline, this is performed by a voting classifier on six consecutive repetitions. This measure thus assesses the overall system performance and may be considered as an indication of the perceived usability, although it is incomplete [24].

### Experiment description

In order to compare both devices, two different experiment conditions were tested: sitting on a chair and walking at 3 km/h on a treadmill, which is a convenient speed for subjects. The ambulatory condition was considered to detect whether the devices have similar relative performances when realistic movement artefacts are produced. It also assesses if the recording systems are fixed enough. To train classifiers and to assess the entire system for each condition separately, each session was composed of one training set and one test set of 25 randomly chosen trials (around 12 minutes for each session). The total duration of the experiment per device was around one hour and a quarter (including breaks and data checking). Recordings were performed on different days for a given subject in a random order.

Eight healthy male and one healthy female subjects participated in this experiment with age between 24 and 34 years old. During the experiment, a 20-inch screen (refreshing rate = 60 Hz) in both conditions was placed at about 1.5 meter in front of the subject for the P300 experiment. Subjects were healthy and did not have any known locomotion-related or P300 disturbing diseases or handicap. All procedures were approved by the Université Libre de Bruxelles Internal Review Board and complied with the standards defined in the Declaration of Helsinki.

### Statistical analysis

In order to detect whether the Emotiv Epoc headset is competitive with respect to the medical device, our statistical assessment is only focusing on comparisons between both devices under each condition excluding cross condition analysis. Indeed, the null hypothesis *H*_0_ assumes that the Emotiv headset is the best device or is equivalent. Then, we are looking for evidence of rejecting it meaning that it is likely to be outperformed.

As applied in [24], although our design follows a repeated measure analysis of variance (ANOVA) for each performance measure [25,26], the omnibus *F* test was not performed. Actually, the omnibus ANOVA *F* test is not a necessary condition to control family-wise error rate (FWER) whatever the applied *post-hoc* tests [26,27]. In this case, the degrees of freedom are spent for somehow useless statistical tests, i.e. tests that do not really correspond to the research question. Furthermore, omnibus *F* test might show no significance while some of the underlying *t*-tests are significant leading to a decrease of power and an overall more conservative test. The important information to remind is that researchers can not continue running different statistical analyses until they obtain the results they desire as FWER quickly inflates.

Instead of the widely used procedure, we thus defined only a limited amount of *a priori* comparisons by applying the prescription of [24,26,27]. First, we defined all the pairwise comparisons. Thereby, we performed the standard paired *t*-tests, whose single assumption is data normality, with a standard alpha level of 5%. Given that those comparisons equal the degrees of freedom, we make sure to control FWER inflation without any further adjustments, which leads to a much more powerful test.

However, obtaining significant results is not enough and effect-size is at least as important [24,26,28]. Significance only assesses if there is enough evidence to determine whether there is a likely effect between two or more groups. It does not provide information about the size of this effect. If the difference is significant but trivial, the best method is not really outperforming the other ones. The normalized unbiased Hedge’ *g*^∗^ effect-size measure somehow tackles this problem by standardly evaluating this effect and by providing some rules of thumb of how big the effect-size is. For instance, absolute Hedge’ *g*^∗^ values around 0.1 (≤ 0.16), 0.2 (0.17–0.32), 0.5 (0.33–0.55) or 0.8 (0.56–1.2) respectively mean a trivial, small, medium or a large effect according to [29,30].

Furthermore, a single value effect size is not sufficient and a 95% interval should be studied. This helps to provide information about how the current effect-size is a good estimation of the underlying one. Obviously, if more data are used, a more precise interval is provided allowing more reliable conclusions. To compute all these values, Matlab and a neuroscience toolbox were used [28].

## Results and discussion

### P300 Results

**Figure 8:**
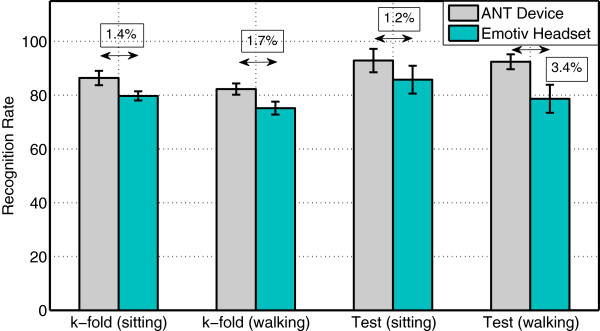
**Results.** This Figure reports average and standard error values of classification rates under sitting/walking conditions for both EEG recording devices as well as *p*-values.

Globally, as shown in Figure 8, the Emotiv Epoc results are not bad at all for such a low-cost system. In fact, the performance is far above the chance level of 25% for each subject, which is consistent with previous studies and responds to old criticisms that this system mainly records muscular and ocular artefacts. Indeed, in this experiment, the elicited P300 response is not synchronized with the gait cycle and thus, the produced Electromyography (EMG) artefacts.

However, as depicted in Figure 8 and in Table 1, the Emotiv headset appears to underperform the ANT system. On a *k*-fold based comparison, *p*-values under sitting and walking conditions are respectively significant at 1.36% and 1.67% with a large effect size indicating relatively large differences in performance. This would mean that the SNR is worse for the Emotiv headset than for the ANT system, which is consistent with [10]. On the test set, both conditions lead to significant results. However, a medium effect is detected under sitting conditions (*p*=1.2*%*) whereas a large effect is again observed under walking conditions (*p*=3.4*%*). Given that effect size intervals are large, the actual value of effect size should be more precisely assessed with a much larger population. Overall, the decrease of performance is coherent with [12] in another context.

**Table 1:** **Hedge *g *results**

Hedgeg^*^	**Lower bound**	**Mean estimate**	**Upper bound**
K-fold (sitting)	0.28	0.95	2.76
K-fold (walking)	0.33	1.00	2.21
Test (sitting)	0.19	0.47	1.81
Test (walking)	0.3	1.05	1.98

Globally, the Emotiv Epoc headset is underperforming the medical device with a medium to large effect size. Confidence intervals were computed by bootstrap as mostly used by the toolbox’s authors [28].

Finally, although the Emotiv Epoc performance is quite creditable especially regarding games, several main points have to be considered. First, the offered performance of the Emotiv headset is lower than the medical-grade system one. For non-critical applications such as games, this low-cost device should suit the customer’s needs. However, in health-related applications or in research, this underperformance could lead to badly performing system that does not work as designed [31]. On the other hand, if researchers are trying to study new potentials in the brain, the lower SNR could make this discovery more difficult [9]. The previous discussion suggests that the design of a new low-cost EEG headset device that disabled people can afford and dedicated to applications needing a highly reliable interface, such as rehabilitation systems, is somehow indispensable.

On the other hand, the presented results were obtained looking at a screen on a treadmill. Although treadmill walking is not that different from free walking, the latter one could lead to a more artefactual environment. It could thus be interesting to investigate whether the differences might modify the conclusions. The Vuzix system or the so-called Google Glass as a substitute of the screen may not be that simple. Indeed, potential interferences between the screen device and the EEG recording system may arise decreasing the overall performance. This should not modify the conclusions as the medical-grade system has already considered this issue. Another aspect would be to study the effect of these devices on the P300 response itself.

#### Other material considerations

**Figure 9:**
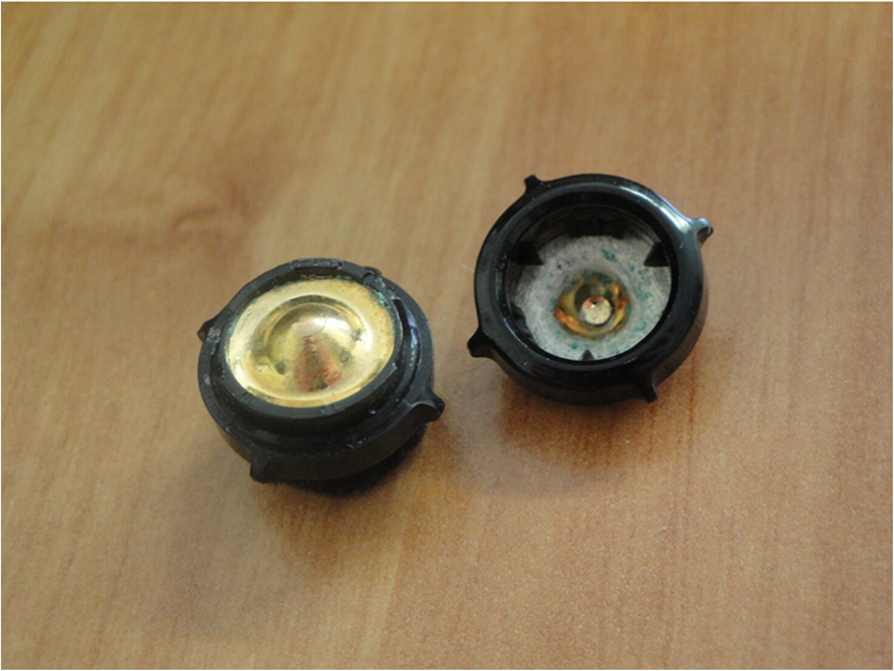
**Oxidation of the electrodes.** The oxidation of the electrode is clearly visible in green. On the back, the non-oxidized electrode has a gold color.

The Emotiv Epoc headset and the ANT device can additionally be compared on other aspects: usability, robustness, cleaning, price, comfort, intrusiveness. In terms of usability, the Emotiv Epoc headset provides a much more user-friendly framework. First, the software user interface is handy and does not require a strong learning. This is much more adapted for end-users than the ANT environment, which was basically designed for researchers and physicians. Then, the headset is easy to place and it can be performed without any exterior assistance whereas the ANT device requires more training and some help at the beginning. However, the positioning of the Emotiv Epoc is somehow imprecise if a specific care is taken, which is typically the case for end-users. This could lead to potential decrease of performance. Although not perfect, this drawback is mitigated in the medical device by the EEG cap. Robustness is a major concern about the Emotiv headset. Indeed, the hardware is basically made of plastics and low-cost components, which results in a fragile headset whereas the ANT device is made of robust textile and cables and the electrodes cupules are made of high quality plastic. In the Emotiv headset, the plastic-based screw thread can easily break up if a particular attention is not brought during each experiment. In case makeshift repairs are not possible, a new headset has to be bought. Moreover, the electrode metallic parts are quickly oxidized even if cleaned at the end of each experiment as shown in Figure 9. After a while, they appear to produce less good signals (during this experiment, all the Emotiv headset electrodes were non oxidized). Moreover, the moss part of all the electrodes is degrading with time and has to be considered as a consumable. All these problems do not happen with the ANT device, which obviously leads to a higher lifespan for the latter system. Thus, the Emotiv headset also requires higher relative operating and maintenance costs. Regarding the cleaning issues, the ANT system has a strong disadvantage. Due to the gel used to decrease the electrode impedance, the subject needs to wash his hair after the experiment. This is not required with the low-cost system as electrode impedance is lowered by a saline liquid. Price is obviously the strongest commercial argument of the Emotiv device. This headset is able to reach satisfying results for an at least 40 times less expensive solution than a medical device. In terms of comfort, people usually reports feeling some pain after one hour of wearing, which is undoubtedly not the case for medical devices. On average, the ANT cap was considered as much more comfortable. Finally, in terms of intrusiveness, the Emotiv Epoc headset was much more broadly accepted. Subjects particularly like the wireless connection (although connection losses may arise especially in closed area) and the design of the low-cost device. However, the ANT device was not designed for ambulatory applications; this latter argument should disappear if a specific ambulatory design were available.

## Conclusions

In conclusion, in this paper, a quantitative comparison of the low-cost Emotiv Epoc headset and the ANT medical/research EEG device is performed based on a standard P300 Brain-Computer Interface. This aims at contributing to get a better picture of how relevant the Emotiv Epoc is [15] and at giving enough information to researchers/end-users who would like to decide in which device they should invest. This work is an extension of a previous preliminary study [13]. When comparing two devices, researchers have to master most of the variables of interest, which depends on what they want to show. In some previous work, the conclusion of the Emotiv Epoc headset underperformance was mainly due to different electrode location and numbers. They could not disentangle this effect from the electrode/amplifier performance. Furthermore, the same process has to be applied on both systems. Given that the reference electrodes are different, a re-referencing after selecting the same electrode locations would give a better picture of their relative performance (in practice, we did not see differences; this could be due to the linear aspect of re-referencing that can be modified by both the spatial filtering and the LDA classifier).

In terms of performance, contrary to some BCI leader criticisms but coherently with previous Emotiv studies, the Emotiv Epoc headset performance is above random and not due to muscular or ocular artefacts. This was supported by far above chance classification rates. While comparing both systems, a large underperformance of the Emotiv device has to be emphasized. On a *k*-fold based comparison, *p*-values under sitting and walking conditions are significant with a large effect size indicating a lower SNR of the Emotiv headset. On the test set, a medium significant effect is detected under sitting conditions whereas a large significant effect is observed under walking conditions. According to these results, the Emotiv Epoc headset is undoubtedly an interesting option. It could be used for non-critical applications such as multimodal games for healthy people or communication and mouse control for disabled users. However, the medium to large underperformance suggests that for critical applications, only a medical-grade EEG recording device should be used. In case researchers can afford having a lot of trials (ERP analysis), it was shown that the Emotiv system may be suitable [9].

In terms of usability, the Emotiv headset has some advantages and disadvantages. It is handy to use, it does only require saline and the investment cost is extremely low compared to a medical-grade system, which is one of the requirements to be widely spread within the disabled people community. On the other hand, subjects are required to learn how to correctly put the headset, connection losses are not infrequent in closed areas, the operating and maintenance costs are relatively higher than in the ANT cap headset and generally, the ANT cap was considered by subjects as more comfortable.

For future work, four main axes can be explored: a larger number of subjects, other BCI paradigms, result durability and the design of a new low-cost EEG headset. Firstly, the precise assessment of the effect size requires much more subjects (around 100 people). Although the observed effect size is coherent with [12], this could help researchers to precisely know what is the relative value of the Emotiv headset.

Secondly, this study focuses on the P300 speller-like system, i.e. inspired from the so-called P300 speller. However, other standard BCI paradigms exist and could be studied to confirm the results presented in this paper. Spontaneous brain signals, e.g. emotion detection and event-related experiments, could be recorded to detect whether similar results can be observed with both devices.

Thirdly, assessing the durability of the Emotiv headset would benefit the scientific community. Indeed, by experience, we know that the saline liquid is evaporating quicker than the ANT gel. This could lead to a drop of performance for applications used on a full-day basis.

Fourthly, given the needs for a higher reliability in critical applications such as rehabilitation or even orthosis/prosthesis control, the design of a new low-cost EEG headset is required. Ideally, this headset should be light, have a large autonomy, have performance closer to a medical system and be relatively cheap.

## Abbreviations

BCI: Brain-computer interface; EEG: Electroencephalography; EMG: Electromyography; ERP: Event-related potential; FWER: Familywise error rate; fMRI: Functional magnetic resonance imaging; fNIRS: Functional near infrared spectroscopy; LDA: Linear discriminant analysis; MEG: Magnetoencephalography; SNR: Signal-to-noise ratio.

## Competing interests

The authors declare that they have no competing interests.

## Authors’ contributions

MD proposed the experiment principles, implemented the OpenVibe pipeline and wrote the first draft. MD and TC proposed the experiment design. TC was in charge of proof-reading. MD, TC, MP and TH participated in the data acquisition. TD and GC supervised the work. All authors read and approved the final manuscript.
